# Genetic and genomic characterization followed by single-step genomic evaluation of withers height in German Warmblood horses

**DOI:** 10.1007/s13353-021-00681-w

**Published:** 2022-01-14

**Authors:** Sarah Vosgerau, Nina Krattenmacher, Clemens Falker-Gieske, Anita Seidel, Jens Tetens, Kathrin F. Stock, Wietje Nolte, Mirell Wobbe, Iulia Blaj, Reinhard Reents, Christa Kühn, Mario von Depka Prondzinski, Ernst Kalm, Georg Thaller

**Affiliations:** 1 Institute of Animal Breeding and Husbandry, Kiel University, Olshausenstr. 40, 24098 Kiel, Germany; 2 Department of Animal Science, University of Göttingen, Burckhardtweg 2, 37077 Göttingen, Germany; 3 Center for Integrated Breeding Research (CiBreed), University of Göttingen, Albrecht-Thaer-Weg 3, 37075 Göttingen, Germany; 4 IT Solutions for Animal Production (Vit), Heinrich-Schroeder-Weg 1, 27283 Verden, Germany; 5 Institute for Animal Breeding and Genetics, University of Veterinary Medicine Hannover, Buenteweg 17, 30559 Hannover, Germany; 6 Institute of Genome Biology, Leibniz Institute for Farm Animal Biology (FBN), Wilhelm-Stahl-Allee 2, 18196 Dummerstorf, Germany; 7 Saxon State Office for Environment, Agriculture and Geology, Schlossallee 1, 01468 Moritzburg, Germany; 8 Faculty of Agricultural and Environmental Sciences, University Rostock, Justus-von-Liebig-Weg 6, 18059 Rostock, Germany; 9 Werlhof-Institut MVZ, Schillerstr. 23, 30159 Hannover, Germany

**Keywords:** Genomic selection, Horse breeding, Withers height, Single-step, German Warmblood breeds

## Abstract

Reliability of genomic predictions is influenced by the size and genetic composition of the reference population. For German Warmblood horses, compilation of a reference population has been enabled through the cooperation of five German breeding associations. In this study, preliminary data from this joint reference population were used to genetically and genomically characterize withers height and to apply single-step methodology for estimating genomic breeding values for withers height. Using data on 2113 mares and their genomic information considering about 62,000 single nucleotide polymorphisms (SNPs), analysis of the genomic relationship revealed substructures reflecting breed origin and different breeding goals of the contributing breeding associations. A genome-wide association study confirmed a known quantitative trait locus (QTL) for withers height on equine chromosome (ECA) 3 close to *LCORL* and identified a further significant peak on ECA 1. Using a single-step approach with a combined relationship matrix, the estimated heritability for withers height was 0.31 (SE = 0.08) and the corresponding genomic breeding values ranged from − 2.94 to 2.96 cm. A mean reliability of 0.38 was realized for these breeding values. The analyses of withers height showed that compiling a reference population across breeds is a suitable strategy for German Warmblood horses. The single-step method is an appealing approach for practical genomic prediction in horses, because not many genotypes are available yet and animals without genotypes can by this way directly contribute to the estimation system.

**Supplementary Information:**

The online version contains supplementary material available at 10.1007/s13353-021-00681-w.

## Introduction

Genomic selection was first proposed 20 years ago by Meuwissen et al. (2001). About 10 years later, advances in molecular genetic technologies and bioinformatics have enabled the implementation of routine genomic evaluation and selection. In the main livestock species, genomic selection is widely applied, whereas in horses, it is still a scarcely used breeding tool. However, the potential of genomic selection in sport horse breeding is high due to the long generation interval (Haberland et al. 2012). The availability of sufficiently large reference populations for the most important breeding goal traits is a major limiting factor for developing genomic selection in horses. Among other parameters such as heritability of the target traits and genetic relationship, the size of the reference population is crucial for the reliability of, and thus, the benefit from genomic breeding values (Meuwissen et al. 2001; Goddard 2009; Van den Berg et al. 2019). In sport horse breeding, where many populations are too small to establish a sufficiently large reference population, collaborative approaches and the development of a reference population across genetically linked breeds are highly reasonable. In dairy cattle, international cooperations were also successfully used to increase the number of animals in the reference population and enable genomic selection in smaller populations (Lund et al. 2016). In order to meet this requirement, a joint project of five German Warmblood breeding associations and partners from science and industry was launched in 2017. The aim of this project is to implement genomic selection in horses by establishing a suitable reference population across genetically linked populations of distinct breeds of Warmblood horses.

To verify the approach and develop the genomic evaluation system, it was decided to use a reference trait as a starting point, similar to milk performance traits in the first application of genomic prediction in dairy cattle. Due to its clear definition, objective measurement, and known genetic characteristics, withers height was chosen for this purpose. In the literature, heritability estimates for withers height of 0.72 for Franches-Montagnes horses (Signer-Hasler et al. 2012), 0.53 for German Warmblood horses (Tetens et al. 2013) and 0.49 for Hanoverian Warmblood horses (Stock and Distl 2006) were reported. A genome-wide association study with 1077 Franches-Montagnes horses revealed two trait-influencing genomic regions (quantitative trait locus (QTL)) on an equine chromosome (ECA) 3 and ECA 9 for withers height (Signer-Hasler et al. 2012). In particular, the QTL on ECA 3 localized near the *LCORL* (ligand-dependent nuclear receptor corepressor-like) gene has later been confirmed in other studies and horse populations (Metzger et al. 2013; Tetens et al. 2013). Furthermore, the *LCORL* gene is known to play a significant role in body size in a wider range of species, including for example humans (Lango Allen et al. 2010) and cattle (Pryce et al. 2011).

A single-step approach for estimating genomic breeding values has been proposed to make optimal use of available data (Aguilar et al. 2010; Christensen and Lund 2010). With this method, all available genotype, phenotype, and pedigree information can be employed simultaneously (Legarra et al. 2014). Not only the performance information from the genotyped but also from the non-genotyped animals is directly included. All existing information can thus be used optimally and not only genotyped animals contribute to the reference population. Since systematic SNP genotyping is not yet common in the horse, the situation with far more non-genotyped than genotyped animals is likely to persist for a longer time period even in those populations interested in implementing genomic selection. Therefore, the use of single-step methodology can be seen as particularly relevant and straightforward for this species (Mark et al. 2014; Stock et al. 2016).

The objective of this study was to genetically and genomically characterize withers height and to develop a system for estimating genomic breeding values for withers height in German Warmblood horses using the single-step approach and a reference population comprising horses from five distinct breeds. For this purpose, preliminary data reflecting the overall structure of the reference population were evaluated.

## Material and methods

### Dataset

For this study, 2157 mares born between 1997 and 2015 from five different German Warmblood horse breeds were both, genotyped, and phenotyped. The distribution of breeds in the dataset was as follows: 1001 mares from Holstein (HOL), 453 from Oldenburg (OL), 100 from Oldenburg International (OS), 427 from Trakehner (TRAK), and 176 from Westfalian (WESTF) studbook. The two Oldenburg breeding associations were considered separately, as they have a different focus with OS predominantly concentrating on the breeding of show jumping horses and OL putting more emphasis on dressage ability. Selection of horses contributing to the reference population was based upon two criteria: (i) a low level of preselection (which was achieved by selecting mares instead of stallions) and (ii) the current genetics of German riding and sport horses should be represented as comprehensive as possible. Therefore, from a larger number of broodmares, those with the lowest pedigree relationships were selected. Genomic data were made available through molecular genetic analyses of genome-wide single nucleotide polymorphisms (SNPs) using commercially available SNP arrays. The GGP Equine 70 k BeadChip® (Neogen/Illumina) containing 65,157 SNPs was used for genotyping of 193 mares, in the following referred to as the first cohort. The remaining 1964 mares (second cohort) were genotyped using the GGP Equine Plus BeadChip® (Neogen/Illumina) which became available more recently and included 6790 additional SNPs, so in total 71,947 SNPs.

Quality control and filtering were performed separately for each cohort using the software PLINK v1.90b6.4 (Purcell et al. 2007). Only autosomal SNPs were considered in the subsequent analyses. Genotyping results were filtered based on Illumina’s GenCall score and applying a threshold of 0.15, which resulted in a mean call rate higher than 0.95 of those SNPs kept for analyses. Markers with a minor allele frequency less than 0.01 and a genotyping rate less than 90% as well as SNPs with a significant deviation from Hardy–Weinberg-Equilibrium (*p*(*χ*^2^) < 0.001) were discarded. Additionally, duplicate SNPs (*n* = 55) were removed. After quality control, the numbers of remaining SNPs were 58,562 for the first cohort and 62,070 for the second cohort. To generate a homogeneous SNP dataset, all animals were imputed to a uniform level using the program Beagle 5.0 (Browning et al. 2018); thus, the additional SNPs on the GGP Equine Plus BeadChip® are also available for the first cohort.

**Fig. 1:**
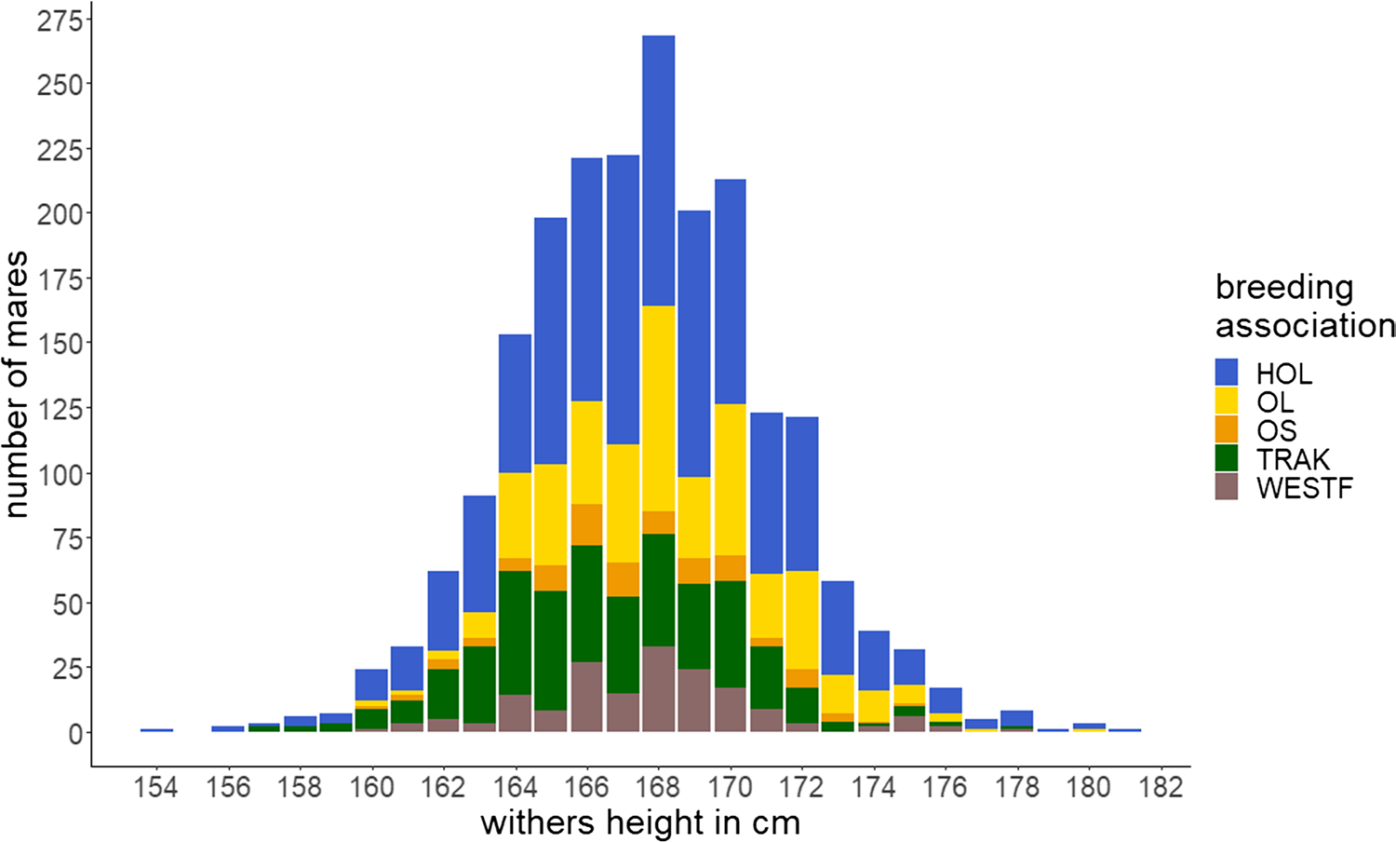
Distribution of withers height for 2113 mares. *HOL*  Holstein horse breed (*n* = 982), *OL* Oldenburger horse breed (*n* = 444), *OS* Oldenburger International horse breed (*n* = 98), *TRAK* Trakehner horse breed (*n* = 416), *WESTF* Westfalian horse breed (*n* = 173)

The final data set used for further analyses included 2113 mares (HOL (*n* = 982), OL (*n* = 444), OS (*n* = 98), TRAK (*n* = 416), and WESTF (*n* = 173)) and 62,070 SNPs. Pedigree information contained three ancestral generations, resulting in a total pedigree size of 14,632 individuals. In particular, the selected mares descended from 608 sires with 1 to 82 offspring (mean 3.5) and from 2006 mares with 1 to 3 offspring (103 mares with 2 offspring, 2 mares with 3 offspring). Table 1 gives an overview on the numbers of sires and dams overall and by breeding association. The most recent measurements of withers height were for 1991 mares from evaluations at studbook registration (SBR) and for 122 mares from supplementary data recording in connection with the mare performance test (MPT). Measurements were taken between 2014 to 2020, when the mares were on average 4.4 ± 2.6 years old. Withers height ranged from 154 to 181 cm with a mean of 167.6 ± 3.5 cm. Figure 1 and Table 1 give more detailed information on the distribution of the investigated trait.

**Table 1:** Distribution of the 2113 mares in the reference population by breeding association (*HOL* Holstein horse breed, *OL* Oldenburger horse breed, *OS* Oldenburger International horse breed, *TRAK* Trakehner horse breed, *WESTF* Westfalian horse breed) and across paternal and maternal progeny groups and descriptive statistics for withers height

Breeding association	*n*	Sires	Dams	Mean	SD	Min	Max
HOL	982	243	933	167.8	3.6	154	181
OL	444	148	424	168.3	3.1	160	180
OS	98	70	91	167.4	3.1	160	175
TRAK	416	145	389	166.6	3.4	157	178
WESTF	173	97	170	167.7	3.1	160	178
Total	2113	608	2006	167.6	3.5	154	181

Mean, standard deviation (SD), minimum (Min), and maximum (Max) were given in centimeters

### Genomic analyses

To examine the suitability of a joint reference population across German Warmblood breeds, the SNP-derived genomic population structure among the 2113 mares was analyzed. First, a principal component analysis (PCA) of the realized genomic relationship matrix across the different subpopulations was conducted using GCTA 1.91.6 beta1 (Yang et al. 2011). Second, using PLINK (Purcell et al. 2007), the linkage disequilibrium (LD; *r*^2^ according to Hill and Robertson 1968) and allele frequencies were determined for the individual breeding associations. Subsequently, a genome‐wide association scan for loci affecting withers height was performed to confirm the major QTL for withers height mapped on ECA 3 (Signer-Hasler et al. 2012; Metzger et al. 2013; Tetens et al. 2013). Therefore, a genome-wide association study (GWAS) was conducted with a leave-one-chromosome-out (LOCO) approach in GCTA (Yang et al. 2011) and the following mixed linear model:

$$y_{\mathrm{ijk}}=\mu+\mathrm{age}\;{\mathrm{class}}_{\mathrm i}+\beta\times{\mathrm{SNP}}_{\mathrm j}+g^-+e_{\mathrm{ijk}}$$

where *y*_ijk_ is the phenotype (mares’ withers height; *k* = 1, …, 2,113); *μ* is the overall mean; age class_i_ represents the fixed effect of the ith age class at measurement (*i* = 1, 2, 3, 4, 5), the first age class contains mares younger than 3 years, the second class includes mares between 3 and 4 years old, the third between 4 and 5 years old, the fourth between 5 and 6 years old, and the fifth group includes all mares older than 6 years old; *β* is the effect of the candidate SNP; SNP_j_ represents the SNP genotype indicator variable (*j* = 0, 1, 2; number of the respective target alleles of the SNP); *g*^−^ is the additive genetic effect based on a genomic relationship matrix calculated using all SNPs except those on the chromosome where the candidate SNP is located; and *e*_ijk_ is the random residual effect. To partition the variance onto different chromosomes and the SNPs identified as significant, a genomic relationship matrix was built separately for the 31 autosomes and the significant SNPs in the GCTA software (Yang et al. 2011). Variance components were estimated using the restricted maximum likelihood analysis. In addition, a conditional association study was performed for the data set. Therefore, single highly associated SNPs were included as fixed effects in the LOCO analysis.

### Genomic prediction

For the estimation of genomic breeding values, a single-step approach was applied. Therefore, pedigree, genotype, and phenotype information were combined for a joint evaluation in an animal model with the BGLR package (Pérez and de los Campos 2014) in the R statistical software (R Core Team 2019). Phenotypes were only available for the genotyped mares (*n* = 2113). First, the numerator relationship matrix (**A**) based on the pedigree information (*n* = 14,632) and the realized genomic relationship matrix (**G**) based on the genotyped animals (*n* = 2113) were built and used to generate a combined relationship matrix (**H**) for all individuals (Legarra et al. 2009). For this approach, the 14,632 horses can be divided into two groups. The first group with non-genotyped horses (*n* = 12,519) and the second group with genotyped horses (*n* = 2113). The inverse of the combined matrix can be computed as (Aguilar et al. 2010; Christensen and Lund 2010):

$${\mathbf{H}}^{-1}={\mathbf{A}}^{-1}+\left[\begin{array}{cc}0& 0\\ 0& {\mathbf{G}}^{-1}-{\mathbf{A}}_{22}^{-1}\end{array}\right]$$

where **A**^−1^ is the inverse of the pedigree relationship matrix for all individuals; **G**^−1^ is the inverse of the genomic relationship matrix for genotyped individuals; and ${\mathbf{A}}_{22}^{-1}$ is the inverse of the pedigree relationship matrix for all genotyped individuals. The single-step model can be written as:

$$\mathbf{y}=\mathbf{X}\mathbf{b}+\mathbf{Z}\mathbf{a}+\mathbf{e}$$

where **y** is the phenotype vector (mares’ withers height); **X** is a design matrix for the effects of age class; **b** is the corresponding vector of fixed effects; **Z** is a design matrix for the random animal effects; **a** is the corresponding vector of additive genetic random effects; and **e** is the random residual effect. To improve the computation speed, the matrix **H** can be transformed such that the random animal effects become independent (Waldmann et al. 2008). To this end, a Cholesky decomposition was used, which led to an equivalent animal model to obtain genomic predictions in a single step:

$$\mathbf{y}=\mathbf{X}\mathbf{b}+{\mathbf{F}}_{\mathrm{a}}{\mathbf{c}}_{\mathrm{a}}+\mathbf{e}$$

where ${\mathbf{F}}_{\mathrm{a}}={\mathbf{Z}\mathbf{H}}^{1/2}$ and ${\mathbf{c}}_{\mathrm{a}}={\mathbf{H}}^{-1/2}\mathbf{a}$. The reliabilities of the individual genomic breeding values were computed with the following equation:

$${\mathrm{r}}^{2}=1-\frac{\mathrm{PEV}}{{\upsigma }_{\mathrm{a}}^{2}}$$

where PEV is the prediction error variance of the breeding values and ${\sigma }_{a}^{2}$ is the additive genetic variance, both estimated in BGLR (Pérez and de los Campos 2014).

## Results

The present data set was used to verify whether a reference population across breeds could be applied for genomic selection in German Warmblood horses. For this purpose, the genomic relationship was analyzed. Afterwards, this reference population was used to check if a known signal for withers height could be found on ECA 3. Furthermore, a genomic breeding value estimation was performed using a single-step approach to determine whether acceptable reliabilities for withers height could be achieved.

### Principal component analysis

**Fig. 2:**
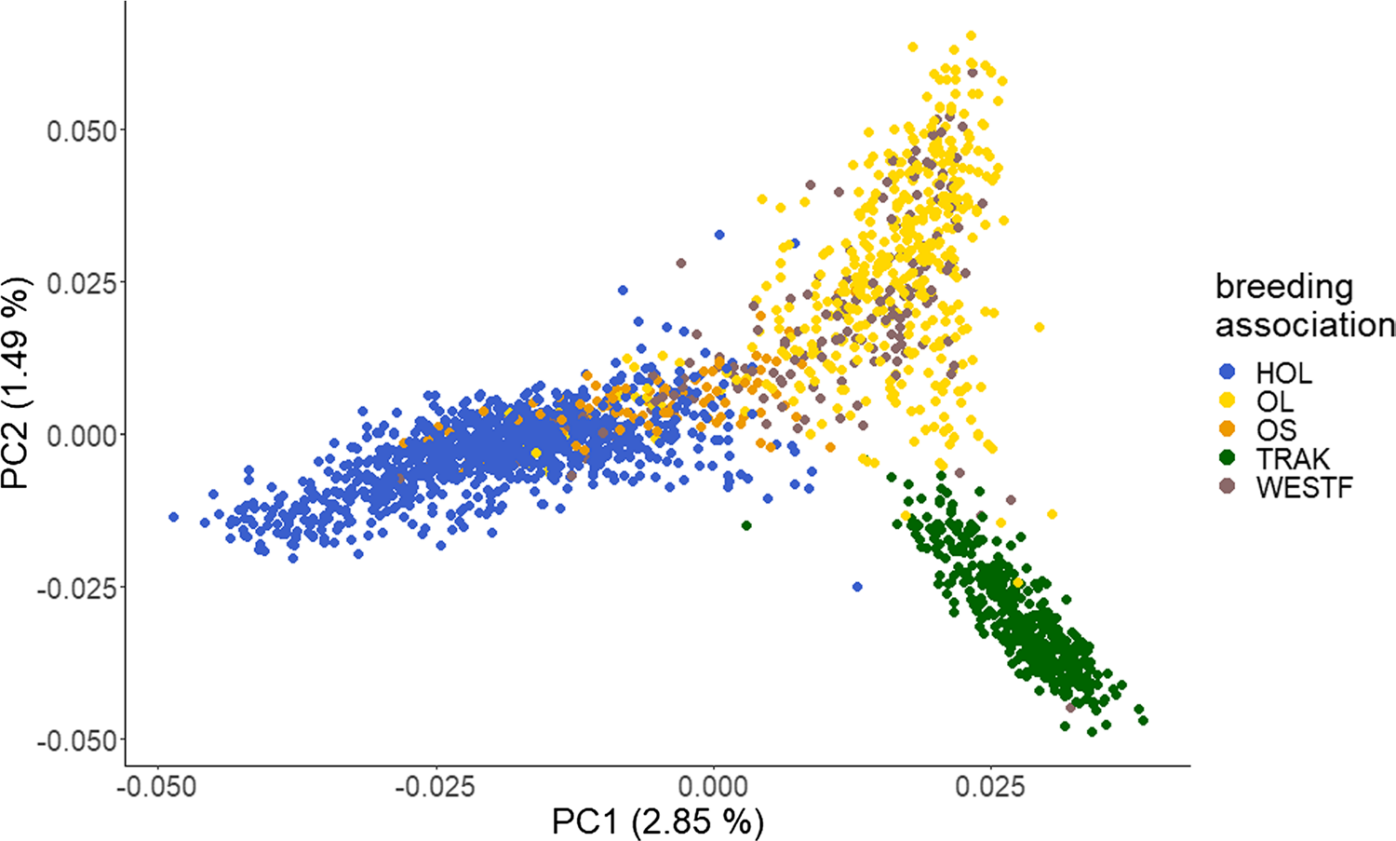
Results of the principal component analysis for 2113 mares. *HOL* Holstein horse breed (*n* = 982), *OL* Oldenburger horse breed (*n* = 444), *OS* Oldenburger International horse breed (*n* = 98), *TRAK* Trakehner horse breed (*n* = 416), *WESTF* Westfalian horse breed (*n* = 173)

The first two eigenvectors of the genomic relationship matrix were used to visualize the relationship at the genomic level. Figure 2 shows the results of the principal component analysis for the 2113 mares. The first principal component (PC1) explained 2.85% and the second principal component (PC2) explained 1.49% of the variation in the data. The PCA revealed three different clusters for the five breeds HOL, OL, OS, TRAK, and WESTF. Substantial overlap was found for OL and WESTF. HOL overlapped with OS along PC1 and PC2. TRAK displayed the most distinct separation. Analyses of the LD (measured as *r*^2^) and allele frequencies underlined the results described above, as the breeds within the clusters showed similar values. This is exemplarily shown for the top associated SNPs in Supplementary table S1.

### GWAS for withers height

**Fig. 3:**
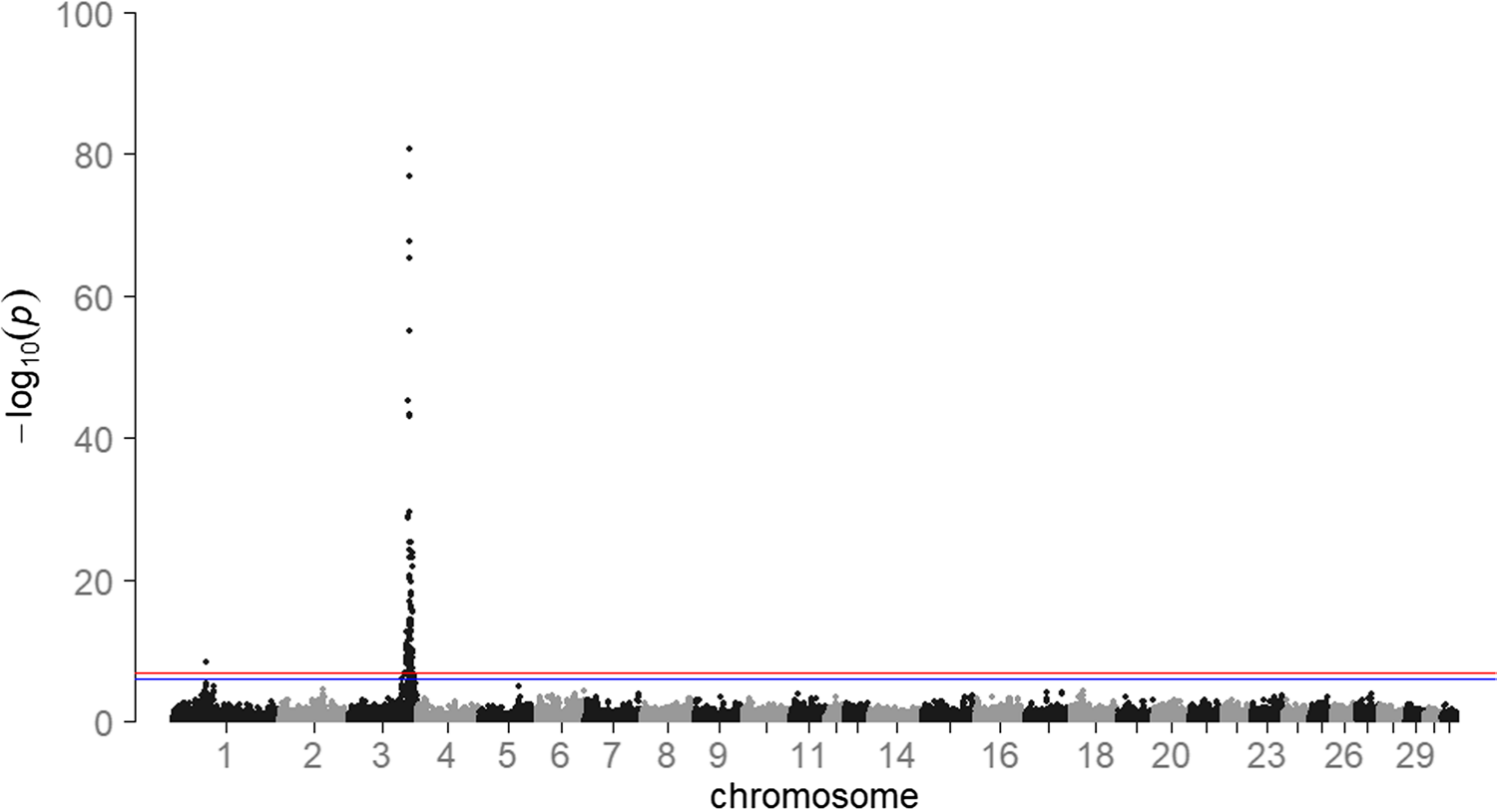
Results of the genome-wide association study for withers height for 2113 mares. *HOL* Holstein horse breed (*n* = 982), *OL* Oldenburger horse breed (*n* = 444), *OS* Oldenburger International horse breed (*n* = 98), *TRAK* Trakehner horse breed (*n* = 416), *WESTF* Westfalian horse breed (*n* = 173). The blue line represents the significance threshold of *P* = 0.01. The red line represents the significance threshold of *P* = 0.05

**Fig. 4:**
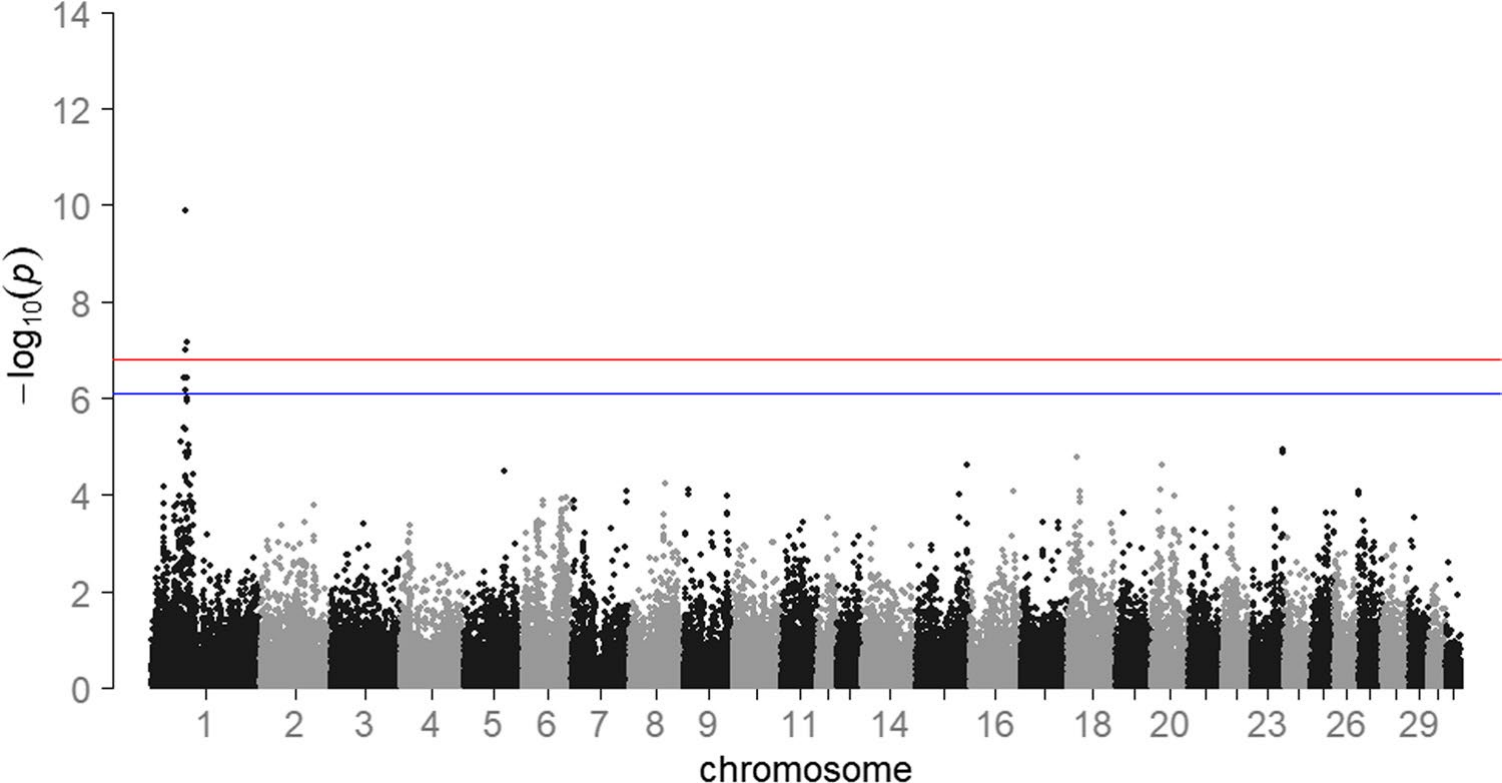
Results of the conditional genome-wide association study for withers height for 2113 mares. *HOL* Holstein horse breed (*n* = 982), *OL* Oldenburger horse breed (*n* = 444), *OS* Oldenburger International horse breed (*n* = 98), *TRAK* Trakehner horse breed (*n* = 416), *WESTF* Westfalian horse breed (*n* = 173). The blue line represents the significance threshold of *P* = 0.01. The red line represents the significance threshold of *P* = 0.05

The GWAS in 2113 mares revealed a single genome-wide significant signal on ECA 3, with the three SNPs BIEC2_808543 (base pair position (EquCab 2.0) 105,547,002 bp), BIEC2_808500 (105,363,241 bp), and BIEC2_808466 (105,163,077 bp) showing the highest negative decadic logarithms of the empirical *p*-values (-log_10_(*p*) values; Fig. 3). The SNP BIEC2_808543, which is highest associated with withers height, is located near the *LCORL* gene and had a minor allele frequency of 0.49. On ECA 1, the SNP BIEC2_23633 (56,208,092 bp) reached the significance level of *P* ≤ 0.05. Analyses of the SNP effects indicated that a few SNPs are strongly associated with the expression of withers height in horses while many SNPs can only explain a minor part of the genetic variance for the trait. The three SNPs on ECA 3 mentioned above together explained 22.8% of the variance. By including the SNP BIEC2_808543 as a fixed effect in the LOCO analysis, the significant signal on ECA 3 dropped completely, only the peak on ECA 1 was above the significant threshold (Fig. 4).

### Genomic breeding values for withers height

**Fig. 5:**
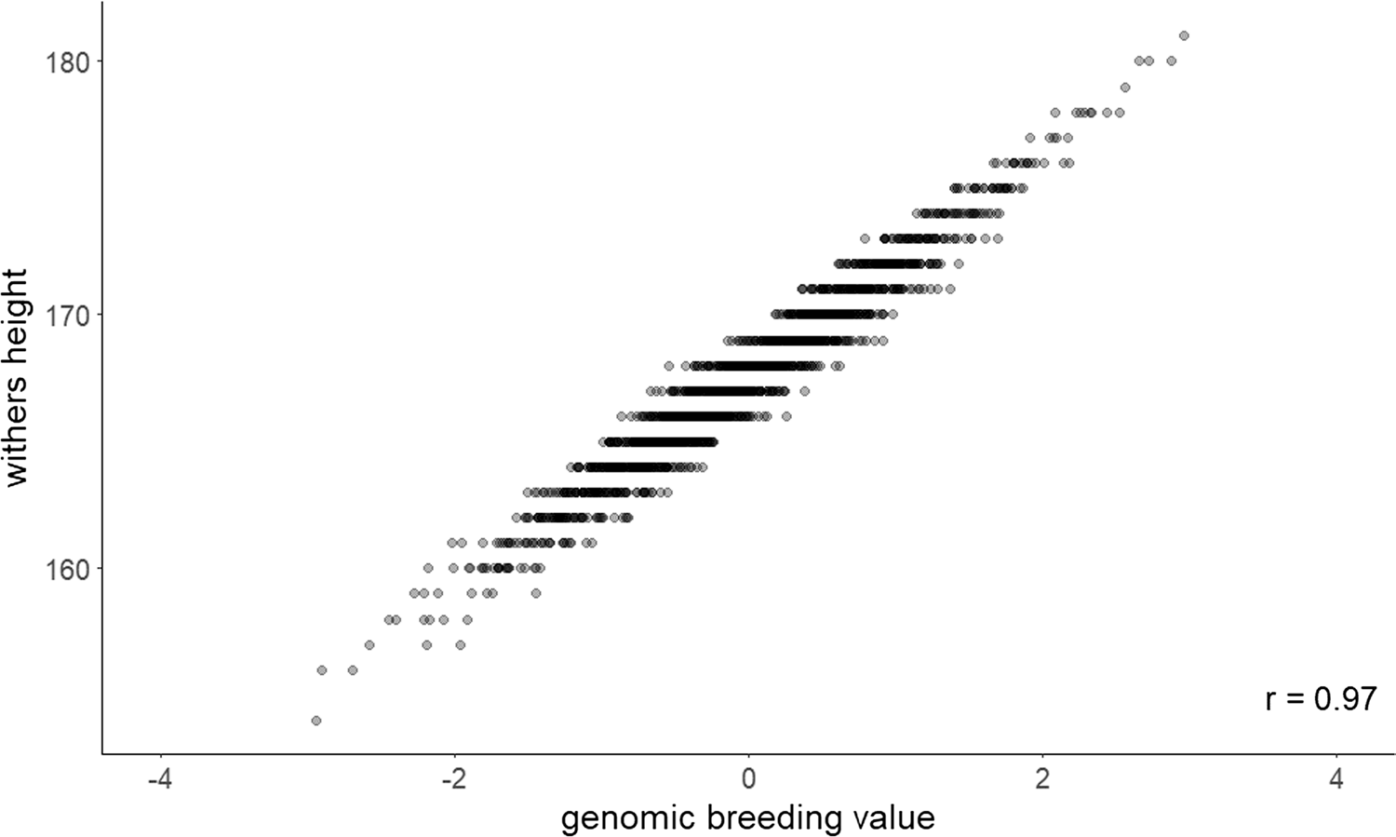
Correlation between genomic breeding value and withers height for 2113 mares. *HOL* Holstein horse breed (*n* = 982), *OL* Oldenburger horse breed (*n* = 444), *OS* Oldenburger International horse breed (*n* = 98), *TRAK* Trakehner horse breed (*n* = 416), *WESTF* Westfalian horse breed (*n* = 173)

**Fig. 6:**
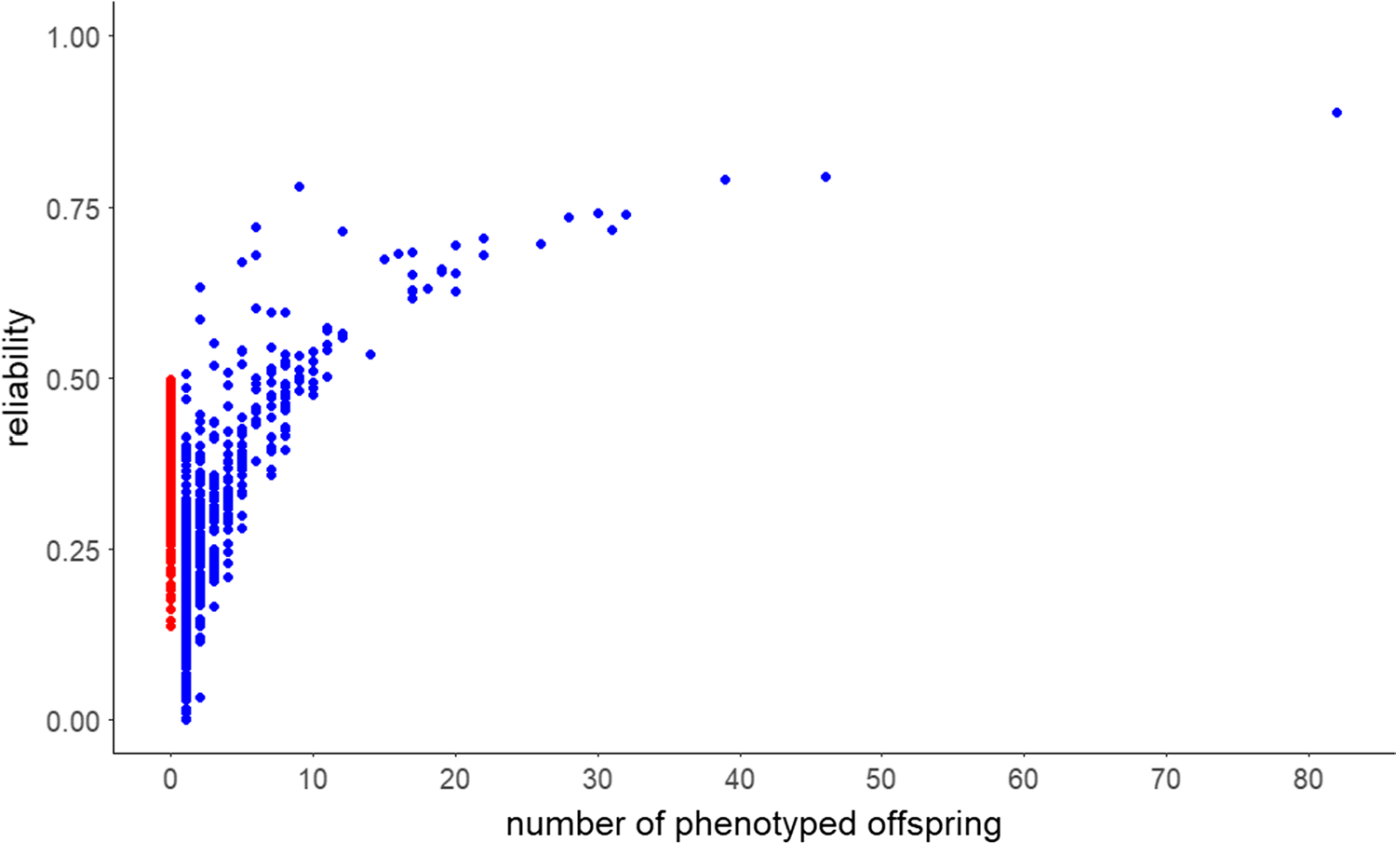
Reliabilities for the genotyped and phenotyped mares (*n* = 2113; red points) and the sires of these mares (*n* = 608; blue points) grouped according to the number of phenotyped offspring

Variance component estimation was done for all 14,632 horses in the pedigree using the transformed matrix H, resulting in a value of 3.95 for the additive genetic variance and a value of 8.62 for the residual variance, and, thus, a heritability of 0.31 with a standard error (SE) of 0.08. The genomic breeding values estimated in this study ranged from − 2.94 to 2.96, with the majority of horses having a value close to 0. The genomic breeding values and the underlying phenotype showed a correlation of *r* = 0.97 for the 2113 mares (Fig. 5). The realized reliabilities (*r*^2^) for the genomic breeding values are shown in Table 2. Figure 6 gives an additional overview of the reliabilities for the genotyped and phenotyped mares and the sires of these mares grouped according to the number of phenotyped offspring.

**Table 2:** Realized reliabilities (*r*^2^) for the genomic breeding values for withers height for 2113 mares

(Sub)sample	*n*	Mean	Min	Max
All horses in the pedigree	14,632	0.13	0	0.89
Horses without phenotype an genotype	12,519	0.09	0	0.89
Horses with phenotype and genotype	2113	0.38	0.14	0.50

## Discussion

In this study, 2113 mares with 62,070 SNPs and data for withers height were available. Data was contributed by five different breeding associations which were not equally represented in the present reference population. Instead, breeding associations with a large total number of broodmares were stronger represented in the reference population than breeding associations with only a small number of broodmares.

### Genomic relationship and potentials and limitations for building a joint reference population

The data set consisted of data from five different horse breeding associations, all of them being subpopulations of the German Warmblood horse. In Germany, conventional breeding values for dressage and jumping performance are routinely estimated jointly for all Warmblood horse breeds. Thus, a joint reference population is the obvious solution to guarantee a fast implementation of genomic selection, especially because most of the subpopulations are much too small to build their own reference population. In dairy cattle breeding, several studies have shown that establishing a joint reference population across related breeds can increase the reliability of genomic breeding values, especially for small breeds (Brøndum et al. 2011; Heringstad et al. 2011; Zhou et al. 2014). However, each breeding association has its own breeding goal and characteristics. On the other hand, there is a growing effort to record new traits (e.g., linear and health traits) using standardized protocols across the different breeding associations.

Figure 2 shows that horses are genomically similar across breeds. Therefore, horses from the five breeding associations and subpopulations used in this study can safely be regarded as one large cohort. Horses from breeding associations with a focus on similar characteristics, e.g., aptitude for a certain equestrian discipline, are most closely related on the genomic level. This is most probably due to the shared use of appropriately qualified sires approved for more than one breeding association. HOL and OS, which both put a strong emphasis on jumping ability in their breeding program, were more related to each other than to OL, WESTF, and TRAK. OL and WESTF, on the other hand, put more weight on the suitability of horses for dressage; this was also reflected in a close relationship at the genomic level. TRAK horses showed comparatively little genomic relationships to the horses registered by the other associations, with a closer relationship to OL and WESTF than to HOL and OS. This result can be explained by the fact that TRAK is the only horse breed bred in accordance with the principles of pure breeding, existing as a partially closed population since 1732. In TRAK, only introgression of English and Arabian Thoroughbred, Shagya, and Anglo-Arabian as breeding animals is allowed (Teegen et al. 2009). These breeds are also used in many other Warmblood breeding associations to refine their horses in a certain direction. In addition to TRAK, the HOL breed also has a closed studbook. On the mare side, it is strictly closed. Currently, no non-Holstein mares can be registered in the studbook. On the stallion side, the breeding is also characterized by the use of English Thoroughbreds and the Arabian horse breed. The use of stallions with foreign blood is minimized. Only a few foreign sires that show exceptional performance in sport and/or in breeding are approved (Roos et al. 2015). The other three breeding associations (OL, OS, and WESTF) are characterized by a more open breeding program and allow the use of foreign sires and dams from other Warmblood breeds, e.g., from one another and from HOL and TRAK.

### Withers height as a reference trait

In sport horse breeding, there are many traits that are difficult to capture or are strongly subjective. Since the success of genomic breeding value estimation and selection strongly depends on the accurate recording of the trait (Daetwyler et al. 2010), withers height is particularly suitable as a reference trait as it is a well-studied as well as an easily and objectively measurable trait. Height, like many other quantitative traits in livestock breeding, is genetically determined by a few SNPs with major and many SNPs with small effects (Hayes and Goddard 2001). In this study, one previously reported genomic region including an important QTL for withers height in horses was confirmed. We identified the SNP BIEC2_808543 on ECA 3 as the SNP highest associated with withers height. In a study of 214 Hanoverian horses by Metzger et al. (2013), the same SNP was significantly associated with withers height with a similar minor allele frequency of 0.45 (vs. 0.49 in the present study). The results of the GWAS confirmed the assumption that there is a genetic variant close to the *LCORL* gene that plays a major role in the expression of withers height in horses (Signer-Hasler et al. 2012; Metzger et al. 2013; Tetens et al. 2013). The association signal could be identified consistently across the study populations. To gather further statistical evidence for the causality of the QTL on ECA 3, a conditional association analysis was conducted (Cohen-Zinder et al. 2005). When including the top SNP (BIEC2_808543) as a fixed effect, no peak was observed above the significance threshold on ECA 3. This suggests that there is only one QTL responsible for withers height in this region. Besides the expected signal on ECA 3, a single SNP on ECA 1 was also significantly associated with withers height. This peak also persisted and got even more pronounced in the conditional GWAS meaning that there is additional genetic variation due to this chromosome. Signer-Hasler et al. (2012) reported that a large fraction of the variance for height in horses could be explained by ECA 1, although they did not find any significantly associated SNPs in this area. A further QTL on ECA 9, also found in the latter study, could not be identified with the present dataset.

The analysis of variance revealed a heritability of 0.31 (SE = 0.08) for the 2113 mares when using the combined relationship matrix, whereas the SNP-based heritability was higher (0.50; SE = 0.05) and more similar to the estimates reported in the literature. Signer-Hasler et al. (2012) estimated a SNP-based heritability of 0.72 for 1077 Franches-Montagnes horses using the Equine 50 k BeadChip® (Illumina). Withers height of the horses ranged from 145 to 165 cm. Tetens et al. (2013) also used the Equine 50 k BeadChip® (Illumina) and calculated a heritability of 0.53 for 782 German Warmblood stallions with a withers height of 158 to 176 cm. In a study by Stock and Distl (2006), a heritability of 0.49 (SE = 0.02) was determined for 20,768 Hanoverian horses with a withers height ranging between 143 and 185 cm.

The objective of this study was to quantify the reliability for first genomic breeding values for the reference trait withers height. For this purpose, a single-step procedure was used. In horse breeding, this is the method of choice because the information from non-genotyped horses can also be incorporated directly and also information from genotyped horses (which are not phenotyped). Especially in the initial phase of genomic selection, this is an advantage, because not many genotypes of horses are available yet (Mark et al. 2014; Stock et al. 2016). Unfortunately, the full potential of this method could not be exploited here because only phenotypes of genotyped horses were available. With the present data set, genomic breeding values for the 2113 mares could be estimated with an average reliability of 0.38. Running a classical BLUP without genomic data yielded in average reliabilities of 0.35 for the same animals. Thus, reliabilities do not increase much by including genotype information. Advantages would possibly be more pronounced, if none phenotyped horses that were genotyped were included in the data, which was however not done in this study. Anyway, the moderate reliability of 0.38 for the genomic breeding values was within the expected range and is comparable to the initial phase of genomic breeding value estimation in dairy cattle breeding for equivalent traits (Luan et al. 2009; VanRaden et al. 2009). Considering all animals in the pedigree, the highest reliability estimates for the genomic breeding values were achieved for non-genotyped and non-phenotyped sires with several genotyped and phenotyped daughters. One sire was represented by 82 offspring in the reference population which resulted in the most reliable breeding value (maximum reliability of 0.89; cf. Figure 6). As expected, the daughters of this sire had the highest reliabilities among the genotyped horses with a mean value of 0.45. It should be noted that many non-genotyped but also some genotyped horses had a reliability of the genomic breeding value close to zero. The latter was observed for mares with genotypes and phenotypes from all five breeding associations and is probably due to the fact that phenotypes were only available for genotyped horses in the reference population, and, furthermore, could be attributed to the selection of mares for the reference population. Here, care was taken to ensure that the animals were as unrelated as possible to each other, thereby, aiming to establish a reference population that represents almost the entire gene pool. However, as a result, relatedness within and between the populations was low with only a small number of common sires. Further enlargement of the current reference population should be done following a selection strategy which puts more focus on close enough relationship structures, e.g., by choosing more mares descending from stallions that were used as sires in more than one subpopulation. In a simulation study by Plieschke et al. (2016), selective genotyping of daughters of certain bulls increased the reliability of predicted breeding values. In dairy cattle breeding, the number of genotyped reference bulls with daughter proofs has been shown to be the most important parameter in determining the reliability of genomic predictions for selection candidates (Goddard and Hayes 2009). A targeted genotyping of stallions from the current breeding population and measurement of their withers height would be useful to further increase the reliability of the calculated genomic breeding values. This is important because low reliability can limit the genetic gain, especially for young animals that are not progeny tested (Thomasen et al. 2014).

In general, our study demonstrates the benefits of using genomic information in horse breeding and shows that using a joint reference population is a suitable strategy for German Warmblood horses. However, the reference population should be further enlarged. Particular attention will be given to the effects of the different subpopulations in further studies aiming to optimize the structure of the reference population. This will, in turn, result in better reliabilities of the genomic breeding values and, thus, increase the acceptance of the horse breeders, which is important for a successful implementation for genomic selection.

## Supplementary Information

Below is the link to the electronic supplementary material.

Supplementary file1 (DOCX 16.8 KB)

## Data Availability

Not applicable.
