# Supplementary material for: Genetic and genomic characterization followed by single-step genomic evaluation of withers height in German Warmblood horses
Source: J Appl Genet. 2022 Jan 14;63(2):369–78. doi: 10.1007/s13353-021-00681-w (PMC8979901; doi:10.1007/s13353-021-00681-w)
Supplement: Supplementary file 1 — Supplementary file1 (DOCX 16.8 KB) [file 13353_2021_681_MOESM1_ESM.docx]

**Table S1 Analyses of the allele frequencies and linkage desiquilibrium of the 2113 mares in the reference population by breeding association (*HOL* Holstein horse breed, *OL* Oldenburger horse breed, *OS* Oldenburger International horse breed, *TRAK* Trakehner horse breed, *WESTF* Westfalian horse breed) for the three top associated SNPs on ECA 3.**

| **SNP** | **Breeding association** | **A1** | **A2** | **MAF** | **r^2^** | | |
| --- | --- | --- | --- | --- | --- | --- | --- |
|  |  |  |  |  | **BIEC2_808543** | **BIEC2_808500** | **BIEC2_808466** |
| BIEC2_808543 | HOL | A | G | 0.43 | - | 0.75 | 0.49 |
|  | OL | G | A | 0.44 | - | 0.60 | 0.75 |
|  | OS | A | G | 0.40 | - | 0.39 | 0.52 |
|  | TRAK | G | A | 0.34 | - | 0.42 | 0.69 |
|  | WESTF | G | A | 0.47 | - | 0.49 | 0.70 |
| BIEC2_808500 | HOL | C | A | 0.37 | 0.75 | - | 0.38 |
|  | OL | C | A | 0.46 | 0.60 | - | 0.48 |
|  | OS | C | A | 0.27 | 0.39 | - | 0.28 |
|  | TRAK | A | C | 0.48 | 0.42 | - | 0.38 |
|  | WESTF | C | A | 0.40 | 0.49 | - | 0.41 |
| BIEC2_808466 | HOL | G | A | 0.40 | 0.49 | 0.38 | - |
|  | OL | G | A | 0.37 | 0.75 | 0.48 | - |
|  | OS | G | A | 0.44 | 0.52 | 0.28 | - |
|  | TRAK | G | A | 0.29 | 0.69 | 0.38 | - |
|  | WESTF | G | A | 0.38 | 0.70 | 0.41 | - |

Single nucleotide polymorphism (SNP), minor allele (A1), major allele (A2), minor allele frequency (MAF), linkage disequilibrium (measured as r^2^).
